# Cloning and Overexpression of Strictosidine β-D-Glucosidase Gene Short Sequence from *Catharanthus roseus* in *Escherichia coli*

**DOI:** 10.15171/apb.2019.076

**Published:** 2019-10-24

**Authors:** Ahmed Saeed Arafa, Amany Elsayed Ragab, Abdel-Rahim Sayed Ibrahim, Wael Saad Abdel-Mageed, Mahmoud Emam Nasr

**Affiliations:** ^1^Pharmacognosy Department, Faculty of Pharmacy, Tanta University, Tanta, Egypt, 31527.; ^2^Molecular Biology Department, Genetic Engineering and Biotechnology Research Institute, Sadat City University, Sadat City, Egypt, 32897.; ^3^Genetics Department, Faculty of Agriculture, Beni-Suef University, Beni-Suef, Egypt, 62511.

**Keywords:** *Catharanthus roseus*, *Escherichia coli*, Strictosidine-β-D-glucosidase, Enzyme assay, Overexpression

## Abstract

***Purpose:*** Strictosidine-β-D-glucosidase (SGD) is considered as a key enzyme in the production of bisindole alkaloids in *Catharanthus roseus*. The present study illustrated the production of a short sequence of this enzyme in *Escherichia coli* without codon optimization.

***Methods: ***Strictosidine-β-D-glucosidase (*sgd*) gene short sequence (1434 bp), which lacks the conserved sequence KGFFVWS and the localization peptide sequence at the C-terminal, was amplified from cDNA of *C. roseus* leaves, cloned and expressed in *Escherichia coli*. The activity of the produced protein in cell free lysate was tested using total alkaloid extract of *C. roseus* leaves.

***Results:*** HPLC and LC-MS analysis of the assay mixture revealed the disappearance of the strictosidine peak.

***Conclusion:*** SGD short sequence can be produced in *Escherichia coli* in active form without codon optimization.

## Introduction

The majority of terpenoid indole alkaloids (TIAs) are confined to the dicotyledons occurring most frequently in Apocynaceae and to a lesser extent in Loganiaceae and Rubiaceae families.^1^ *Catharanthus roseus* (L.), a member of Apocynaceae family, produces more than 130 monoterpenoid indole alkaloid which have several medical properties.^2-4^ No other single plant species was reported to produce such a wide array of complex alkaloids.^5^ Alkaloids from *C. roseus* have hypotensive, sedative, anti-cancer, anti-diabetic and stomachic activities.^6-8^ *C. roseus* is a source of very important anti-tumor agents, vincristine and vinblastine, in its leaves and the well-known antihypertensive alkaloids, serpentine and ajmalicine in the root part.^9-11^

**Figure 1:**
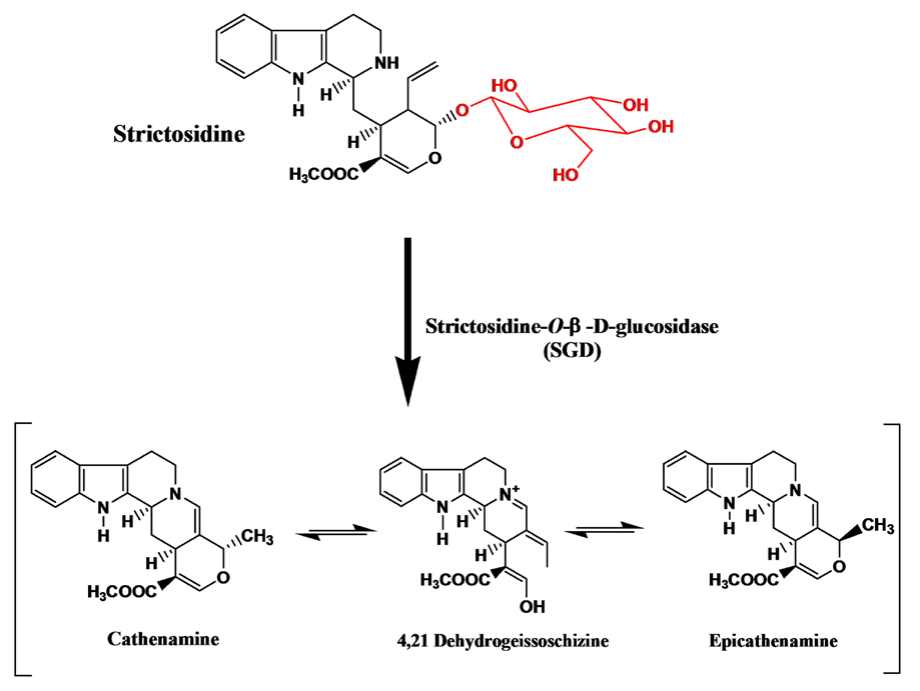
Deglucosylation of strictosidine by strictosidine-β-D-glucosidase.^2^

The biosynthesis of TIAs in *C. roseus* involves more than 35 known intermediates and 30 enzymatic steps.^2^ Strictosidine is the key precursor for all TIAs and results from the strictosidine synthase (STR) catalysed condensation of secologanin and tryptamine.^12-14^ Strictosidine is then deglucosylated by strictosidine-β-D-glucosidase (SGD) to form a highly reactive intermediate (Figure 1) which is considered the first step in creating biosynthetic diversity in all TIAs producing plants.^15,16^

*Catharanthus roseus* has different isoforms of β-glucosidases,^17^ among these is SGD which was first purified to apparent homogeneity from cell suspension culture of *C. roseus*as a high molecular mass protein complex with high affinity for strictosidine.^18^ Earlier localization study using novel staining method reported that SGD is most likely associated with the endoplasmic reticulum (ER). This is consistent with the presence of three signal peptides in the gene sequence, one of them is located at the *C*-terminal (KKQKY) and the other two (SRL, SHL) are internal signals.^19^ Another localization study was conducted using green fluorescent protein imaging experiments to report the nuclear localization of SGD, which is consistent with the presence of a *C*-terminal nuclear localization signal (537-KKRFREEDKLVELVKKQKY-555).^20^

A codon optimized full length synthetic *C. roseus sgd* was expressed in *Escherichia coli*to study its activity against various substrates. *C. roseus* SGD was found non-stereoselective because it turns over strictosidine and vincoside. Consequently, stereoselectivity of biosynthesis of TIAs is dependent on STR, but not SGD. SGD acts with high efficiency on strictosidine analogues with methyl substituent in the indole ring and with low efficiency on analogues that contain pentynyl ester.^21,22^

The yield of vinca alkaloids is not high (0.00025% from *C. roseus* dry leaves for vinblastine). Therefore, synthetic methods were used to produce these compounds which is tedious.^23-28^ *In vitro* use of the biosynthetic enzymes is another option. However, in plants, compartmization of enzymes and the possible need for transport proteins in some reactions would limit the *in vitro* use of enzymes. To date, there are no studies for *in vitro* production of vinca alkaloids, vincristine and vinblastine, in bacteria.

The full length SGD (555 amino acids, 1668 bp) has the highly conserved sequences T(L/I)FHWD and KG(Y/F)(Y/F)(V/A)WS of plant β-glucosidases. It was amplified from *C. roseus* cDNA using degenerate primers based on these conserved sequences and was produced in *Saccharomyces cerevisiae*.^19^ In SGD, the sequences TLFHWD is located towards the *N* terminal, while the sequence KGFFVWS is close to the *C* terminal of the protein. Caros009426.1 is an SGD that lacks the conserved sequence KGFFVWS and the signal peptide sequence for localization (KKRFREEDKLVELVKKQKY). To determine whether or not, the localization is mandatory for the activity, we expressed Caros009426.1 as a short sequence of SGD (478 amino acids) in *E. coli*without codon optimization.

## Materials and Methods

### Apparatus and chemicals

Kendro Biofuge Primo R centrifuge (Hamburg, Germany) was used for centrifugation under cooling. SDE-PLAS horizontal electrophoresis unit connected to Consort E865 electrophoresis power supply (Turnhout, Belgium) and a Syngene ultraviolet (UV) transilluminator (Cambridge, UK) was used for running and visualisation of agarose gels. Running sodium dodecyl sulfate polyacrylamide gel electrophoresis (SDS-PAGE) gels was performed using a Hoefer SE 245 mighty small vertical gel electrophoresis unit (Holliston, Massachusetts, USA). Polymerase chain reactions (PCR) were done using a Biometra T-Gradient thermoblock (Göttingen, Germany). Sonication of proteins in lysis buffer was carried out using a Cole Parmer 4710 series ultrasonic homogenizer (Chicago, USA). DNA sequencing was performed using Applied Biosystems “ABI Prism 3700” Capillary-Sequencer (California, USA). High performance liquid chromatography (HPLC) measurements were carried out using Agilent Technologies 1260 Infinity instrument (California, USA) equipped with G1329B automatic injector, G1311C quaternary pump, and G1314F variable wavelength detector. Liquid chromatography-mass spectrometry (LC-MS) analysis was carried out using XEVO triple quadrupole instrument from Waters Corporation (Milford, Massachusetts, USA). The peaks and spectra were processed using the Maslynx 4.1 software. Chemicals for microbiological media were purchased fromSigma Aldrich (Haverhill, UK), adenosine triphosphate (ATP) was purchased from Oxford (Maharashtra, India), and imidazole was purchased from Merck (New Jersey, USA). Solvents for extraction and HPLC analysis were obtained from Fisher Chemical (Loughborough, UK).

### Bacterial strains, vectors, media, enzymes and kits

*Escherichia coli* DH5α (Invitrogen, Schwerte, Germany) was used for cloning, and *E. coli* BL21 (DE3) (Invitrogen, Schwerte, Germany) was used for protein expression. pET26b (+) vector was purchased from Novagen (Darmstadt, Germany).

Luria-Bertani (LB) media consisted of: 0.5% (w/v) NaCl, 1% (w/v) peptone, and 0.5% (w/v) yeast extract, and sterilized by autoclaving. Agar was added for preparation of standard agar media at 1.5% (w/v). Lysis buffer consisted of: 50 mM NaH_2_PO_4_, 300 mM NaCl, and 20 mM imidazole, pH 7.8.

*Nco*1 and *Xho*1 restriction enzymes, shrimp alkaline phosphatase, and phusion high-fidelity DNA polymerase were purchased from New England Biolabs (Massachusetts, USA). Trizol^®^ reagent, T4 DNA ligase, Thermo Scientific RevertAid H Minus first strand cDNA synthesis kit, and PageRuler unstained protein ladder were purchased from Thermo Fisher Scientific (Ohio, USA). Zyppy^TM^ plasmid miniprep kit, used for plasmid isolation, Zymoclean^TM^ Gel DNA recovery kit, used for DNA purification, and Mix and Go *E. coli* transformation kit were purchased from Zymo Research (Irvine, California, USA). HyperLadder^TM^ 1 kb was purchased from Bioline (London, UK). Protein concentration was determined using Quick Start^TM^ Bradford protein assay kit from Bio-Rad (Washington, USA).

### Total RNA isolation

The plant leaves were ground using liquid nitrogen, then total RNA was isolated from tissue samples (100 mg) using TRIzol^®^ reagent according to the manufacturer’s manual. RNA pellets were resuspended in 40 μL RNase-free water and stored at -80°C.

### Amplification ofsgd-cDNA

First strand cDNA was synthesized using RevertAid H Minus first strand cDNA synthesis kit according to the manufacturer’s manual.

PCR was carried out using phusion high-fidelity DNA polymerase under the following conditions: 98°C for 30 seconds, followed by 35 cycles of: 98°C for 12 seconds, 55°C for 30 seconds, 72°C for 45 seconds, then hold at 72°C for 7 min. The 1473-bp fragment was ampliﬁed with primers (5’-AACCATGGAACACCA CCACCACCACCATATGGGA TCTAAAGATGACCA GTC-3’, forward) and (5’-GGCTCGAGTCACAC ACCATCATCAATA GCATCTC-3’, reverse), that were designed to introduce histidine tag at *N*-terminal, and restriction sites for *Nco*1 and *Xho*1 at the forward and reverse ends of the open reading frame, respectively. Thymine-18 was changed to cytosine in forward primer to decrease primer dimer formation, they encode the same amino acid Asp-6.

### Preparation of pET-26b (+)/sgdconstruct

PCR purified gene and pET-26b (+) vector were double digested with *Nco*1 and *Xho*1 at 37°C overnight. Inactivation of the restriction enzymes was performed at 65°C for 20 min. For plasmid, restriction digestion was followed by dephosphorylation using shrimp alkaline phosphatase and incubation continued at 37°C for 60 min. The digested gene and plasmid were purified and checked on agarose gel electrophoresis. The digested gene was inserted into linearized dephosphorylated vector using 2 units of T4 DNA ligase, 0.4 μL 25 mM ATP, and 2 μL 10x ligation buffer in a total volume of 20 μL. The ligation reactions were assembled on ice and incubated at 16°C overnight then transformed into *E. coli* DH5α using Mix and Go *E. coli* transformation kit. The product of the transformation reaction was plated on solid LB agar containing 50 μg mL-^1^ kanamycin and incubated overnight at 37ºC. Colonies with pET-26b (+)/*sgd* construct were identified using colony PCR. The positive colony was grown in LB medium containing 50 μg mL-^1^ kanamycin at 37°C for 24 h and subjected to plasmid isolation. The presence of the inserted gene was confirmed by restriction digestion. The identity of *sgd* gene was confirmed by DNA sequencing.

### Protein expression

The recombinant plasmid was isolated and transformed to *E. coli* BL21. Overnight cultures of *E. coli* BL21 transformed with pET-26b (+) or pET-26b (+)/*sgd* constructs were diluted 100-fold into fresh LB (2 flasks each contained 500 ml LB) containing 50 μg ml^-1^ kanamycin and incubated at 37°C, 100 rpm until the OD_600_ reached 0.6. Protein expression was induced with isopropyl β-D-1-thiogalactopyranoside (IPTG, final concentration 0.1 mM) and protein production was performed at 18°C for 48 h, 100 rpm. Cells were collected by centrifugation at 4°C (4500 rpm, 45 min), washed with lysis buffer, resuspended in lysis buffer (40 mL for pellet obtained from 500 mL) and lysed through sonication. Cell debris was removed by centrifugation at 4°C (16 000 rpm, 45 min). The resulted supernatants were tested for SGD protein using SDS-PAGE analysis.

### Total alkaloid extraction from leaves of Catharanthus roseus

Fresh leaves of *C. roseus* (45 g) were collected (Genetic Engineering and Biotechnology Research Institute, Sadat City University, Sadat City, Egypt, August 2017), shade dried then ground to fine powder. Dry powder (5 g) was sonicated for 30 min with methanol (MeOH, 400 mL x 3) then filtered. The filtrate was evaporated using rotary evaporator. The residue (600 mg) was dissolved in 0.3 N HCl (200 mL) then extracted with ethyl acetate x3. The aqueous layer was separated, and pH was adjusted to 8 using ammonium hydroxide and extracted with ethyl acetate x3. The ethyl acetate layer was separated and evaporated using rotary evaporator, and the residue (40 mg) was dissolved in HPLC MeOH (5 mL).^29^

### Strictosidine β-D-glucosidase enzyme assay

Assay was performed using cell free lysate of BL21/pET-26b (+)/*sgd* and plant extract. The assay reactions contain 7.5 -15 μg protein and 40 μg alkaloid extract in 5 μL MeOH in a total volume of 100 μL citrate /phosphate buffer (pH 6.0).^30^ The control reaction was prepared using cell free lysate of BL21/ pET-26b (+). The samples were incubated for 1 h at 30°C,^31^ the reaction was terminated by addition of 200 μL MeOH, centrifuged at 11 000 × g for 5 min, and the supernatants were analysed for enzyme activity. The activity of SGD enzyme was tested using HPLC and LC-MS analysis. The HPLC system, described by Hallard et al.^32^ and modified from Pennings et al,^33^ was used for testing enzyme activity. The mobile phase consisted of 7 mM sodium dodecyl sulphate in MeOH (A) and 25 mM NaHPO_4_ in water pH 6.2 (B) with isocratic elution of 63 (A): 37 (B) v/v. The mobile phase was filtered and degassed under vacuum. The analysis was carried out at room temperature using flow rate 1 mL/min on a 4.6 x 250 mm, 5 μm particle size Inertsil^®^ ODS-3 column. The change in alkaloid content was detected at 280 nm. LC-MS analysis described by Brown et al,^34^ was followed with some modification. The analysis was carried out using Acquity UPLC BEH C18 1.7 µm 2.1 × 50 mm column, 0.1% formic acid in water (A), MeOH (B) with a gradient 90-10% A over 30 min, a flow rate of 0.2 mL/min and detected in electrospray ionization (ESI, +ve mode).

## Results and Discussion

### Preparation of pET-26b (+)/sgdconstruct

**Figure 2:**
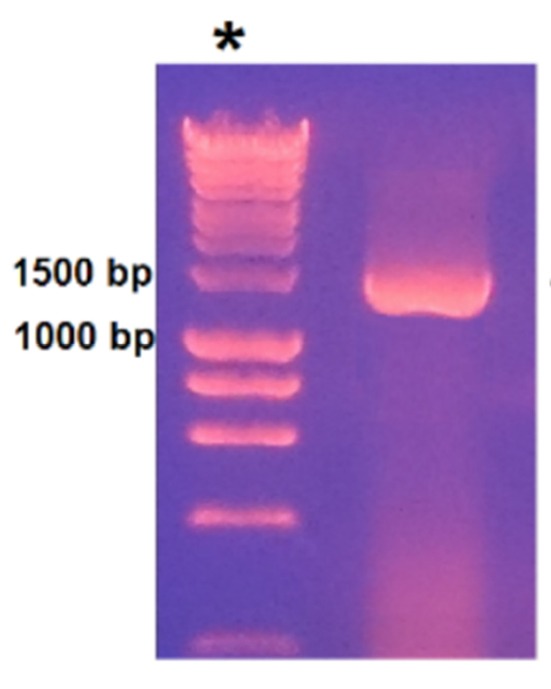
Agarose gel electrophoresis showed amplified strictosidine-β-D-glucosidase short sequence gene(*sgd-sh*). ^*^ HyperLadder^TM^ 1kb. bp: base pair.

**Figure 3:**
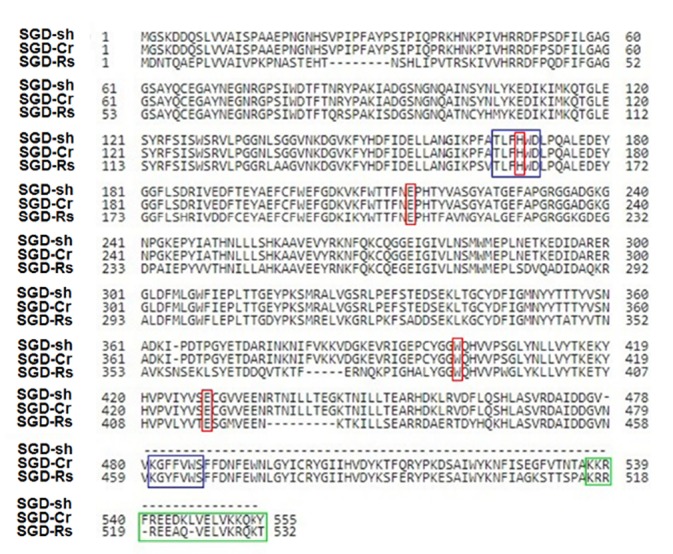
Alignment of amino acid sequence of *C. roseus* SGD short sequence (SGD-sh), *C. roseus* SGD whole sequence (SGD-Cr), and *R.serpentina* SGD (SGD-Rs) using protein Basic Local Alignment Search (blast) tool. Amino acids proposed for catalysis are framed in red; Conserved sequences for plant β-glucosidases are framed in blue; nuclear localization sequence is framed in green.

To PCR-amplify *sgd*, primers were designed using the sequence for Caros009426.1 in the database of the online resource for community annotation of eukaryotes (ORCAE) (http://bioinformatics.psb.ugent.be/orcae/annotation/Catro/current/Caros009426.1).^35^ A PCR fragment of the size 1473 bp (Figure 2) was obtained which is compared to the size of *sgd* gene from *C. roseus* in literature.^19^ The correct insertion of *sgd* gene into pET-26b (+) vector was confirmed by DNA sequencing. The resulted sequence was aligned to *C. roseus sgd* codon sequence (CDS) using nucleotide basic local alignment search tool (blast) (https://blast.ncbi.nlm.nih.gov/Blast.cgi). Beside the intentionally changed nucleotide in the forward primer, there was only one nucleotide difference, Guanine-1320 instead of Adenine-1320 that encodes the same amino acid Leu-440. Barleben et al studied the conserved sites for catalysis in *Rauvolfia serpentina* SGD (SGD-Rs) through site directed mutagenesis.^36^ Alignment of the resulted amino acid sequence of full length *C. roseus* SGD (SGD-Cr), short SGD (SGD-sh) and (SGD-Rs) using protein blast tool (https://blast.ncbi.nlm.nih.gov/Blast.cgi) revealed thattheamino acids proposed for activity in SGD-Rs (His-161, Glu-207, Trp-388, Glu-416) are represented in SGD-Cr by His-169, Glu-215, Trp-400, Glu-428 (Figure 3). The alignment also indicated that SGD-sh lacks the amino acids (479-555) which include the localization sequence and conserved sequence KGFFVWS.

### Protein production

**Figure 4:**
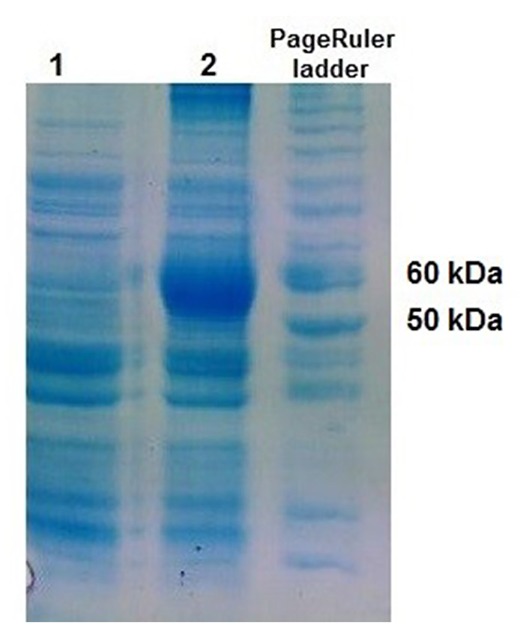
Coomassie blue stained SDS-PAGE of: Lane1, control [*E. coli* transformed with pET-26b (+)] cell lysate, lane 2, cell lysate of *E. coli* transformed with pET-26b (+)/*sgd*construct. kDa: Kilodalton; SDS-PAGE: sodium dodecyl sulfate polyacrylamide gel electrophoresis.

*Sgd-sh* gene was overexpressed in *E. coli* BL21 using IPTG after transformation with pET-26b (+)/*sgd*construct and SGD productionwas confirmed using SDS-PAGE (Figure 4). The calculated molecular weight of the produced SGD protein is 54.7 kDa.

### Short strictosidine β-D-glucosidase assay

**Figure 5:**
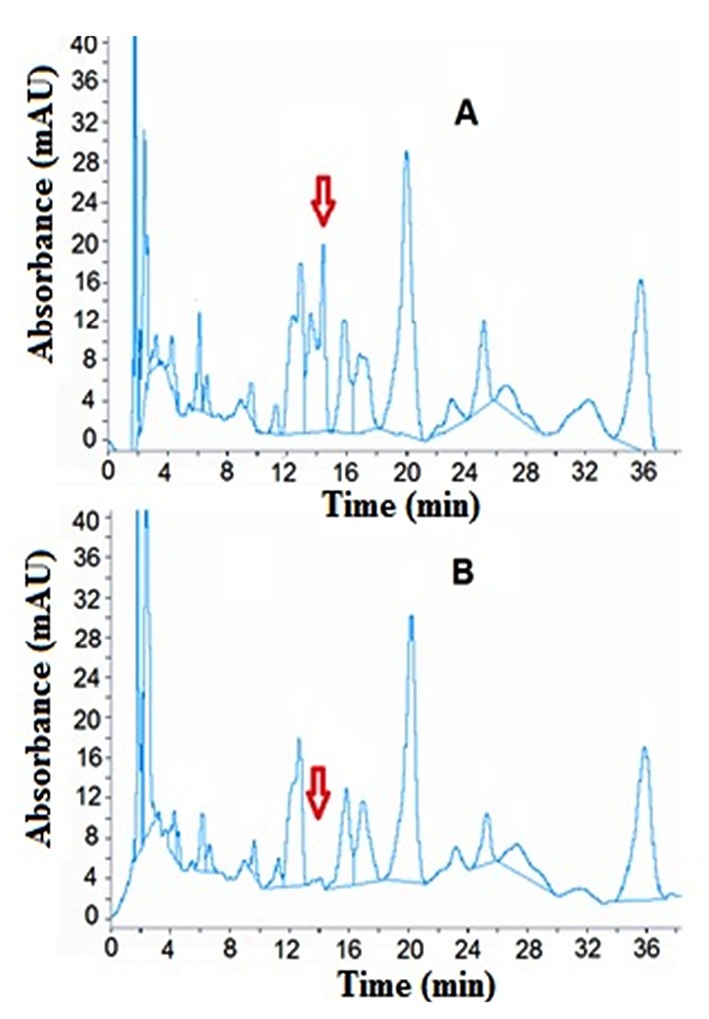
High performance liquid chromatography (HPLC) chromatogram for *C. roseus* alkaloid extract treated with cell lysate from *E. coli*BL21/pET-26b (+)/*sgd*:**A**. control sample with protein produced by *E. coli* BL21/pET-26b (+). **B**. sample treated with 15 μg protein from *E. coli*BL21/pET-26b (+)/*sgd*. mAU: Milli-Absorption Unit.

The activity of SGD-sh in cell lysate was studied using plant alkaloid extract as a substrate and analysed by HPLC. The plant alkaloid extract was used, and not pure strictosidine, to explore the effect of the enzyme on the whole alkaloid content. The end product of strictosidine deglucosylation is cathenamine which is water insoluble,^1^ so we detected the change in strictosidine peak as an indicator of the enzyme activity. It was found that, after incubation of 15 μg of total protein from *E. coli* transformed with pET-26b (+)/*sgd* construct with plant extract for 1 h at 30°C, no new peaks were observed and one peak with retention time 14.4 min disappeared in comparison with control in which the plant extract was incubated with the protein extract of *E. coli* transformed with pET-26b (+) (Figure 5).

**Figure 6:**
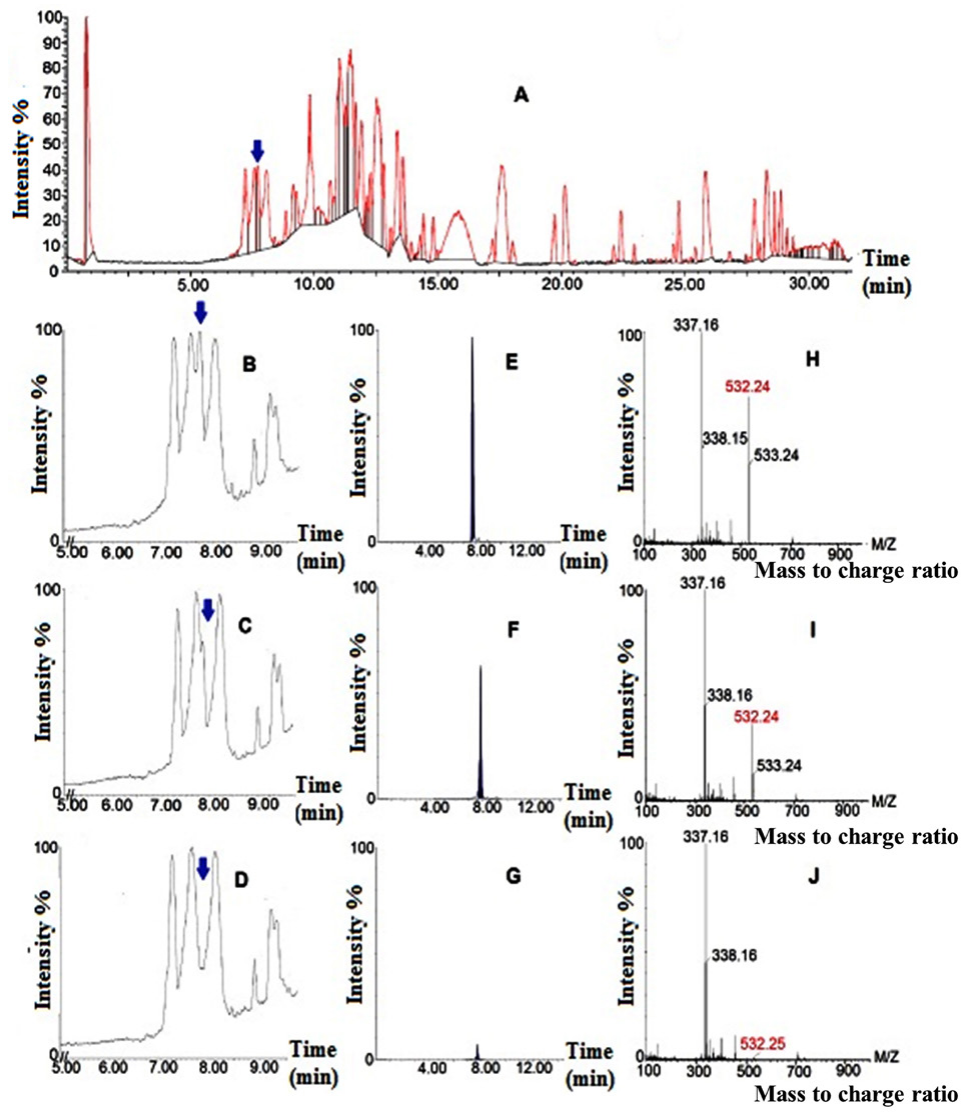
Liquid chromatography-mass spectrometry (LC-MS) analysis of *C. roseus* alkaloid extract treated with cell lysate from *E. coli* BL21/pET-26b (+)/*sgd*.**A:**LC-trace of control reaction with protein produced by *E. coli* BL21/pET-26b (+); **B:**LC-trace of control reactionzoomed in (5- 10 min); **C:**reaction treated with 7.5 μg protein from *E. coli* BL21/pET-26b (+)/*sgd*; **D:**sample treated with 15 μg protein from *E. coli* BL21/pET-26b (+)/*sgd*. **E, F, G:** extracted ion chromatograms of strictosidine ([M + H^+^] *m/z* 532) in B, C and D reactions, respectively; **H, I, J:** mass spectrum at retention time 7.7-7.8 min for E, F and G, respectively.

The glucosidase activity of the *E. coli* cell lysate was confirmed using LC-MS analysis depending on strictosidine mass ([M + H^+^] *m/z* 532). The intensity of strictosidine peak at 7.75 min decreased after incubation of total alkaloid extract of *C. roseus* with 7.5 μg total protein of *E. coli* BL21/pET-26b (+)/*sgd* for 1 h at 30°C, while incubation with 15 μg of the same protein resulted in further lowering strictosidine content to traces (Figure 6, panels C, D, respectively). Control test with protein produced by *E. coli* BL21/pET-26b (+) showed no change (Figure 6, panels A, B). The results indicated that SGD-sh which was obtained without codon optimization and lacking the conserved sequence KGFFVWS and the signal sequence KKRFREEDKLVELVKKQKY, in the cell lysate was active. This result suggests that these sequences have no impact on or not essential for the glucosidase activity. However, that might have an effect on the kinetics and or thermal stability of the enzyme. The previous results indicated that the full-length SGD is associated with the ER or the nucleus due to the presence of the signal peptide sequence, which was confirmed by detecting the deglucosylation product as a precipitate on the plant cell nucleus.^19,20^ The different localization of strictosidine and SGD indicates that the transport of strictosidine by a transport protein to where SGD is located, could be a rate limiting step in the biosynthetic pathway. However, in our study the short SGD was found active in bacterial cell lysate which indicated that intact ER or nucleus is not mandatory for the sugar cleavage step in cell lysate and there might be no need for the transport protein.

## Conclusion

SGD short sequence was found active in bacterial cell lysate after protein expression induction by IPTG without codon optimization of the gene or the use of synthetic gene to adjust the GC ratio to that of the bacterial host. Therefore, it was concluded that the catalytic cleavage of glucose is not connected to nucleus and the bacterial expressing system and environment may compensate the need for transport proteins. This study augments the trend of using bacterial hosts, which are cheap and easy to manipulate, as a mimic to the plant biosynthetic system to produce natural products of plant source. Additionally, the *in vitro* production studies of vinca alkaloids in bacterial hosts may be achievable in the future by overcoming the need for localization of bioconversion reactions and the related transport proteins. SGD coupling with STR and tabersonine 16-hydroxylase (T16H) in expression studies might be promising for vincristine and vinblastine production.

## Ethical Issues

Not applicable.

## Conflict of Interest

There is no conflict of interest.

## Acknowledgments

The authors are very grateful to Assist. Prof. Mustafa Sakr, Dr. Tamer Mesalamy, Dr. Ahmed Salah, and Dr. Mohamed El-Sayed (Genetic Engineering & Biotechnology Institute, Sadat City University, Egypt) for constructive discussions. We acknowledge Dr. Asmaa Abdelhamid (Research fellow at Norwich medical school, University of East Anglia, UK) for English editing.
